# Extramedullary Plasmacytoma of the Larynx Treated by a Surgical Endoscopic Approach and Radiotherapy

**DOI:** 10.1155/2015/951583

**Published:** 2015-06-04

**Authors:** Massimiliano Pino, Filippo Farri, Pietro Garofalo, Fausto Taranto, Andrea Toso, Paolo Aluffi

**Affiliations:** ^1^E.N.T. Department, University “Amedeo Avogadro” of Piemonte Orientale, 28100 Novara, Italy; ^2^E.N.T. Department, A.O.U “Città della Salute-Regina Margherita”, 10126 Torino, Italy

## Abstract

Extramedullary plasmacytoma (EMP) is a rare variant of plasma cell myeloma that affects soft tissues. The head and neck region are the most affected sites, although others have also been described. Herein we report an uncommon case of EMP of the larynx in a 65-year-old male who presented with a history of progressive dysphonia and hoarseness. Laryngeal fiberscopy evidenced a reddish pedicled voluminous mass in the left false cords and ventricle. Microscopic suspension laryngoscopy was performed under general anaesthesia and a 4 W Acublade CO2 Laser was used for transoral resection of the lesion. This was followed by adjuvant radiotherapy, with the widely recommended doses on the supraglottic region, to achieve better local control. Diagnosis of EMP is based on immunohistochemistry and the exclusion of systemic plasma cell proliferative disorders. Diagnosis of solitary EMP can be made only if studies for disseminated disease and X-ray and/or magnetic resonance imaging of the spine, pelvis, femurs, and humerus and bone marrow biopsy are negative. As there are no internationally established guidelines, treatment of EMP is mainly based on consensus of expert opinion.

## 1. Introduction

Plasma cell neoplasms represent a spectrum of diseases ranging from benign conditions, such as monoclonal gammopathy, to malignant entities, such as plasma cell myeloma and plasma cell leukemia [1]. Plasma cell myeloma (multiple myeloma (MM)), a neoplastic proliferation of plasma cells [2], is the most common plasma neoplasm and is characterized by the involvement of the bone marrow at multiple sites [3]. A solitary plasmacytoma is a single localized mass of neoplastic plasma cells occurring in either bone (medullary) or soft tissue (extramedullary). The localized variant may be either the first evidence of generalized myeloma or an independent solitary lesion, characterized by the production of monoclonal immunoglobulins detectable in the serum and/or urine [2].

Most cases of extramedullary plasmacytoma (EMP) are seen in older men (male : female ratio 3 : 1, with a peak incidence in the 50–70-year-old group [4]). Extramedullary plasmacytomas have been reported in various sites in the body, such as the airway passages, gastrointestinal tract, and soft tissues [5]. However, about 80% of extramedullary plasmacytomas occur in the head and neck region [6], mainly in the nasal cavity, paranasal sinuses, or nasopharynx [4], whilst EMP of the larynx is quite rare.

## 2. Case Report

**Figure 1:**
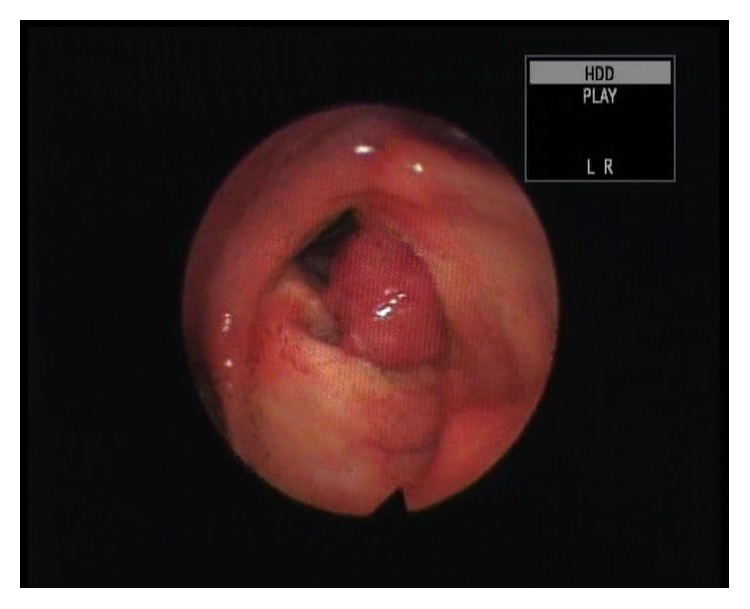
Laryngeal fiberscopy revealed a reddish pedunculated voluminous mass localized in the left false cord and ventricle.

P. L., a 65-year-old male, presented to the Ear, Nose and Throat (E.N.T.) Department of the* Azienda Ospedaliero-Universitaria “Maggiore della Carità*,*”* Novara, Italy. He was complaining of dysphonia that had worsened continuously over a 6-month period, accompanied with a slight dysphagia of a 3-month onset. Laryngeal fiberscopy evidenced a reddish pedunculated voluminous mass in the left false cords and ventricle (Figure 1), without impairment of the airway patency. No lymph node enlargement was detected at neck palpation.

Direct suspension laryngoscopy was performed under general anaesthesia with a binocular microscope having a 400 mm objective lens coupled with a CO2 laser (Acublade SuperPulse 4 W). The lesion was radically resected with CO2 laser, removing the left false cord whilst preserving the left vocal cord. Systemic antibiotics (Cephalosporin) and PPIs were administered to prevent the formation of excessive fibrin and granulation tissue, which might lead to stenosis.

The patient then had adjuvant radiotherapy at a dose of 46 Gy in 24 fractions on the supraglottic region, due to closed tumour-free margins.

**Figure 2:**
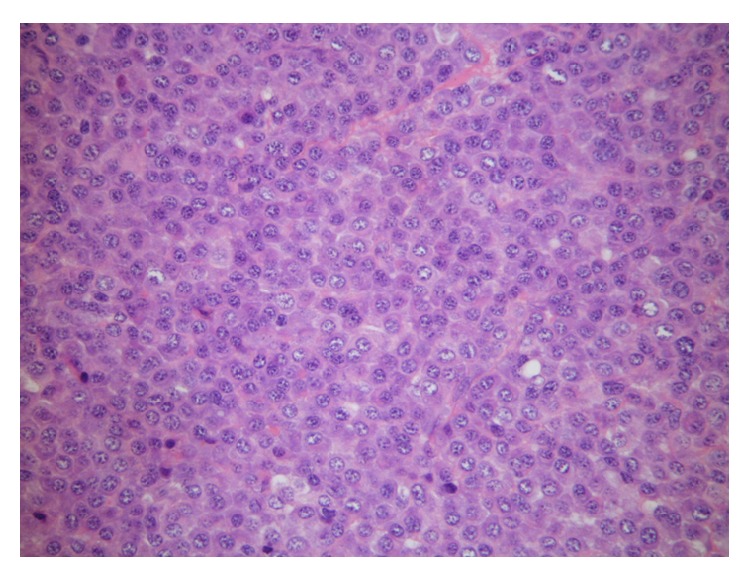
Monomorphic population of atypical plasma cells.

**Figure 3:**
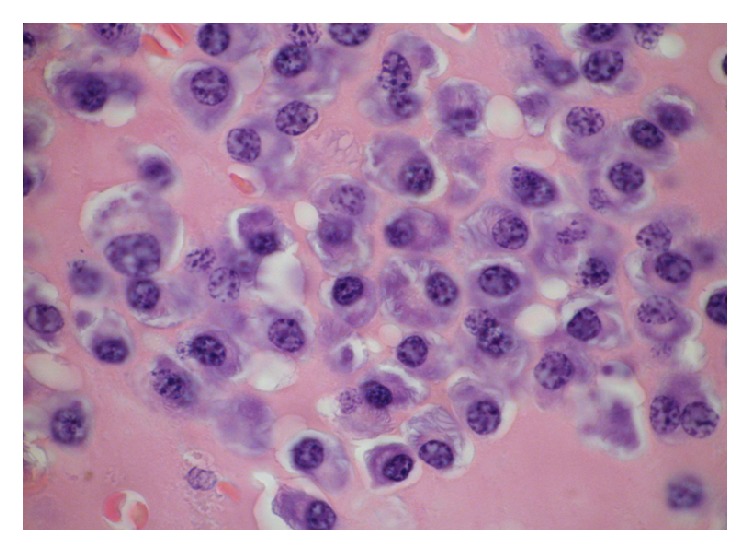
Plasma cells presented eccentric nuclei and atypical cytology (prominent nucleoli, dispersed nuclear chromatin, and high nuclear-cytoplasmic ratio).

**Figure 4:**
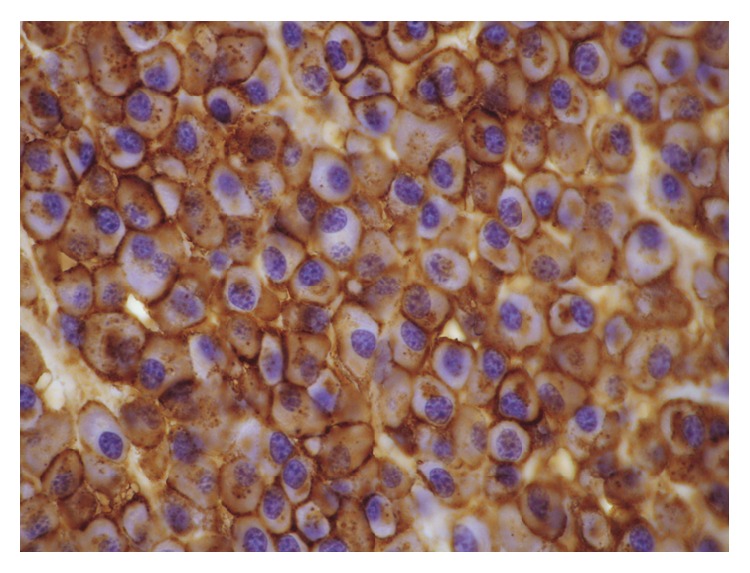
Neoplastic cells showed diffuse membrane positivity for CD138 at immunohistochemical staining.

Histopathology revealed a tumour with a monomorphic population of atypical plasma cells, which had totally or partially substituted the laryngeal architecture (Figure 2). Higher magnificationshowed that plasma cells had eccentric nuclei and atypical cytology (prominent nucleoli, dispersed nuclear chromatin, and a high nuclear-cytoplasmic ratio) (Figure 3). Neoplastic cells showed diffuse membrane positivity for CD138 at immunohistochemical staining (Figure 4).

Both the histopathological and immunohistochemical features were consistent with a diagnosis of extramedullary plasmacytoma. Laboratory examinations (full blood counts, serum chemistry, and serum protein immunoelectrophoresis- (*IEP-*)serum test) were within the normal range. Further systemic investigations, that is, X-ray of the spine, pelvis, femurs, humerus, and bone marrow biopsy, were performed after surgical excision so as to stage the disease. A diagnosis of solitary extramedullary plasmacytoma was made on the basis of the findings.

## 3. Discussion

Although EMP of the larynx is rare, it is on the increase, with 4.5% to 18% of EMP of the head and neck occurring in the larynx [7]. Although the clinical presentation varies depending on the organ involved [8], EMP of the larynx often presents with hoarseness and/or dysphagia [7]. Cervical node metastasis and acute laryngeal obstruction have also been reported [7]. Common sites of laryngeal involvement in order of frequency are the epiglottis, ventricles, the vocal folds, false cords, the aryepiglottic folds, arytenoids, and subglottis [7]. Plasmacytomas in the larynx vary from smooth polypoidal tumours with narrow bases to sessile tumours with wide attachments, which may be red or pale pink.

Diagnosis of extramedullary plasmacytoma is based on the exclusion of a systemic plasma cell proliferative disorders and immunohistochemistry results. On light microscopy, extramedullary plasmacytoma must be differentiated from a reactive plasmacytosis, plasma cell granuloma, poorly differentiated neoplasms, immunoblastic lymphoma, or extranodal marginal zone B-cell lymphoma with plasmacytic differentiation. Plasmacytoid lymphoma has a mixture of lymphocytes and plasma cells. Immunoblastic lymphomas show a cytoplasmic IgM heavy chain and express pan B-cell surface antigen, such as CD19 and CD20 [2].

The diagnosis of plasmacytoma necessitates a detailed evaluation so as to exclude multiple myeloma [4]. Diagnosis of solitary EMP should be made only if studies for disseminated disease are negative. X-ray and/or magnetic resonance imaging of the spine, pelvis, femurs, and humerus, and bone marrow biopsy should also be within the normal range. There should be no signs of serum urine monoclonal protein, anaemia, hypercalcemia, or renal impairment [8]. A prospective study by Schirrmeister et al. has also assessed the accuracy of positron emission tomography (PET) scanning in staging patients with presumed solitary plasmacytoma [9].

There are no generally accepted guidelines for the treatment of patients with EMP and most recommendations remain as consensus of expert opinion [10]. Indeed, due to the rarity of this neoplasm, most studies are retrospective and no randomized trials are available.

There are several strategies which may be adopted, including surgery, radiotherapy, chemotherapy, and combinations of these, but, to date, radiotherapy is the standard treatment for EMP, as it is highly radiosensitive.

Alexiou et al. [11] reviewed 714 cases in literature published between 1905 and 1997: radiation therapy alone was used in 44.3% of cases and surgery alone in 21.9%, with combined therapy (radiation after surgery) in 26.9% of the cases. Liebross et al. [12] reported 19 cases, where all but one had been treated with radiotherapy alone.

To date, although there is no confirmed set dose regimen for radiotherapy, doses from 40 to 60 Gy are recommended [13–15]. Hughes et al. [16] suggested that the optimal radiation dose is dictated by the size of the tumour, with doses in the range of 40 Gy in 20 fractions recommended for tumours <5 cm, whereas 50 Gy in 25 fractions was recommended for tumours >5 cm.

Radiotherapy alone, at a dose of 40 to 50 Gy, has been reported to give 100% local control in plasmacytoma of the vocal cord [15], even if no dose-response relationship has yet been demonstrated. Surgical resection represents a favourable therapeutical option for small EMP of the larynx. Whilst endoscopic laser microsurgery permits a favourable bioexcision of small lesions that may even be considered a definitive treatment if the tumour is resected with histopathological confirmation of wide tumour-free margins, in cases of positive and/or close margins, or in the presence of large lesions that cannot be resected endoscopically, adjuvant RT is a must.

Gorenstein et al. [17] suggested that small tumours could be treated by laryngeal microsurgery and that rarely radical surgery is necessary. Alexiou et al. [11] reported that surgery alone suffices if the tumour is localized and that if complete removal is impossible, surgery followed by radiotherapy is advisable.

According to the accepted recommendations, our patient was given adjuvant radiotherapy after CO2 laser resection to achieve better local control at a dose of 46 Gy in 24 fractions in the supraglottic region. Pathological diagnosis was consistent with laryngeal plasmacytoma. Laboratory examinations (full blood, serum chemistry, and serum protein immunoelectrophoresis) were within the normal range. Further systemic investigations were performed after excision to stage the disease and a diagnosis of solitary extramedullary plasmacytoma was made. No recurrent or disseminated diseases were detected at the 54-month follow-up and, at time of writing, the patient is in remission.

Treatment for local recurrence and progression to MM differs. Local recurrence is to be treated with radiotherapy or surgery, whilst progression to MM necessitates chemotherapy. As extramedullary plasmacytomas of the head and neck may progress with involvement of regional lymph nodes, the use of RT therapy to any adjacent lymph nodes has been recommended [11].

According to Holland et al., the size of the tumour at diagnosis, total serum protein level, and a monoclonal spike observed on serum protein electrophoresis are predictive factors of progression to MM [18], which is observed in 10–40% of cases, mainly within the first 2 years from disease onset [14].

The prognosis of EMP is relatively favourable compared to that of solitary plasmacytoma of bone or MM. In a study on 25 cases, reported by Sunsnerwala et al., the median survival rate was 68 months for EMP [19] and 14.5 for MM arising from EMP [18]. A long follow-up is advisable as EMP may have long time intervals between relapse and progression to MM, even as long as 10 years [20].

In conclusion, diagnosis of extramedullary plasmacytoma is based on the exclusion of a systemic plasma cell neoplasm (MM) and a thorough radiological investigation as aforementioned, with negative results and no signs of renal impairment and even if it has better prognosis than multiple myeloma, it should always be considered malignant or potentially malignant and treated as such. As plasmacytoma of the head and neck region has better prognosis than other sites, the treatment strategy is to be chosen with care, aiming at the preservation of laryngeal function whenever possible. As it is on the increase, we emphasise the need for EMP to be taken into consideration in the differential diagnosis of laryngeal neoplasms.

Moreover, at this point we may also ask ourselves if this increase is also due to dissemination of knowledge on this rare finding. Therefore, further prospective and randomized trials would help, not only to inform but also to clarify the question.
